# Validity and Cross-Cultural Adaptation of the Persian Version of the Oxford Elbow Score

**DOI:** 10.1155/2014/381237

**Published:** 2014-08-26

**Authors:** Mohammad H. Ebrahimzadeh, Amir Reza Kachooei, Ehsan Vahedi, Ali Moradi, Zeinab Mashayekhi, Mohammad Hallaj-Moghaddam, Mehran Azami, Ali Birjandinejad

**Affiliations:** ^1^Orthopaedic Research Center, Ghaem Hospital, Mashhad University of Medical Sciences, Mashhad 91766-99199, Iran; ^2^Massachusetts General Hospital, Harvard Medical School, Boston, MA 02114, USA; ^3^Mashhad University of Medical Sciences, Mashhad 91766-99199, Iran; ^4^Yawkey Center, 55 Fruit Street, Suite 2100, Boston, MA 02114, USA; ^5^Orthopedic Research Center, Emam Reza Hospital, Mashhad University of Medical Sciences, Mashhad 91379-13316, Iran; ^6^Orthopaedic Research Center, Shahid Kamyab Hospital, Mashhad University of Medical Sciences, Mashhad 913791-3316, Iran

## Abstract

Oxford Elbow Score (OES) is a patient-reported questionnaire used to assess outcomes after elbow surgery. The aim of this study was to validate and adapt the OES into Persian language. After forward-backward translation of the OES into Persian, a total number of 92 patients after elbow surgeries completed the Persian OES along with the Persian DASH and SF-36. To assess test-retest reliability, 31 randomly selected patients (34%) completed the Persian OES again after three days while abstaining from all forms of therapeutic regimens. Reliability of the Persian OES was assessed by measuring intraclass correlation coefficient (ICC) for test-retest reliability and Cronbach's alpha for internal consistency. Spearman's correlation coefficient was used to test the construct validity. Cronbach's alpha coefficient was 0.92 showing excellent reliability. Cronbach's alpha for function, pain, and social-psychological subscales was 0.95, 0.86, and 0.85, respectively. Intraclass correlation coefficient (ICC) was 0.85 for the overall questionnaire and 0.90, 0.76, and 0.75 for function, pain, and social-psychological subscales, respectively. Construct validity was confirmed as the Spearman correlation between OES and DASH was 0.80. Persian OES is a valid and reliable patient-reported outcome measure to assess postsurgical elbow status in Persian speaking population.

## 1. Introduction

Clinical outcome measures to evaluate health related quality and function are important in the field of orthopedics [1, 2]. Scoring systems are of two parts that include in one part clinical evaluation and judgment by a skilled observer. The second part is the opinion of patients regarding their health status and this may differ from their physician. Whereas patient satisfaction and well-being are the aim of all therapeutic protocols, relying upon only clinical measures is not enough and so considering the opinion of patients concerning their health status is necessary to standardize medical or surgical decisions [3]. Moreover, another point to consider is that some health status items such as pain or psychosocial characteristics are not completely evaluable by clinical observation. Hence, to compare the efficacy of different treatment protocols with each other, we should put emphasis on joint clinician and patient reported measures, which is why patient reported outcome measures (PROMs) have been introduced [4].

According to a meta-analysis performed by Longo et al., eighteen questionnaires are available to assess the elbow joint and these can be classified into two models [5]. One model is to classify elbow questionnaires into patient or clinician reported or combined forms. Another model is to classify them into elbow-specific or general questionnaires. The disabilities of the arm, shoulder, and hand (DASH), Quick-DASH, musculoskeletal function assessment (MFA), and short musculoskeletal function assessment (SMFA) are examples of the general measures that include items related to the elbow whereas the American shoulder and elbow surgeons (ASES), patient-rated elbow evaluation (PREE), Mayo elbow performance index (MEPI), Liverpool elbow score (LES), and Oxford Elbow Score (OES) are elbow-specific instruments. Each questionnaire is limited to different aspects of the elbow joint making it difficult to select the most comprehensive scoring system.

Currently, the only elbow-specific measure, which has been validated using a high-quality methodology on heterogeneous study populations, is the Oxford Elbow Score (OES) [6]. OES has been validated in few countries. Yet, only the Dutch and Danish versions of OES have been validated and cross-culturally adapted in the literature [7, 8]. In the current study, we aimed to assess the validity and reliability of the Persian translation of the Oxford Elbow Score (Persian OES) in patients having had elbow surgery.

## 2. Materials and Methods

### 2.1. Forward-Backward Translation

After receiving the License Agreement from the University of Oxford (ISIS Innovation Ltd.; ISIS Project number 3737), we used the 10-step forward-backward method following the Wild et al. guideline to translate the OES into Persian [9]. Initially, two independent translators whose mother tongue was Persian translated the original English form of the OES into Persian. Then, an observer compared the two Persian translations and reconciled the two translations. In the next step, a native English speaker who was blind to the original English OES translated the reconciled Persian OES back into English. The back-translated version was then compared to the original OES version by the research team and minor discrepancies were addressed afterwards to prepare the prefinal Persian OES. In a pilot study on 10 patients, we tested face validity and addressed the difficulties in understanding and interpretation. After proofreading, the Persian OES was finalized to be administered in validity and reliability testing. (see Supplementary File in Supplementary Material available online at http://dx.doi.org/10.1155/2014/381237).

### 2.2. Patients

Ninety-two patients with surgery about the elbow participated in the current study and filled out three forms of the Persian version of the OES, DASH, and SF-36. To assess the test-retest reliability, 31 patients were randomly selected to fill the Persian OES again three days after the first visit while refraining from any treatment during these days. After the questionnaire was completed, the patients were instructed to mail it back to us. Our inclusion criteria were having an intervention such as injection or surgery due to a condition about the elbow including trauma, fractures, tendinitis, bursitis, ulnar neuritis, decreased elbow function, pain, and limitation of elbow motion. We included patients if the time elapsed from surgery was more than four weeks. Moreover, all of the patients had to have the ability to read the questionnaire. Patients with simultaneous problems in any region of the body or upper limb which may have influenced the results of the DASH or SF-36 were excluded from the study.

Patients' age ranged from 14 to 77 years (mean ± SD: 40 ± 15). Forty male and 52 female enrolled in the study. Thirty-six patients had less than a high school diploma and the others had a high school diploma and higher. Elbow condition was confined to right elbow in 55 patients and to left elbow in 37 patients. Forty-eight patients (53%) had received cortisone injection for lateral epicondylitis, medial epicondylitis, and tenosynovitis. Eight patients had a history of a previous fracture or dislocation with subsequent surgery while the others had the history of surgery for heterotopic ossification, contracture release, loose body, Tennis elbow, and ulnar nerve anterior transposition.

The study was reviewed and approved by the Committee of Research of the Mashhad University of Medical Sciences (MUMS Research Project number 910078) and all of the patients were informed regarding the research study and signed a consent form.

### 2.3. Psychometrics

To assess the construct validity of the Persian OES, scores of all the three questionnaires were compared with each other using the Spearman's correlation coefficient [10]. We calculated the intraclass correlation coefficient (ICC) to test the reliability of the test-retest and to test how reproducible the questionnaire would be [11]. Also, we used the Cronbach's *α* coefficient to assess the internal consistency across the Persian OES subscales by comparing scores of each question with the other questions [12]. Cronbach's *α* coefficient indirectly shows the extent to which all of the 12 items of the Persian OES measure the same construct.

To validate the Persian OES, we used the Persian version of related scoring systems. Currently, the only available Persian scoring systems related to the elbow, upper extremity, and general health status are the Persian DASH and Persian SF-36.

### 2.4. Oxford Elbow Score (OES)

This is a short 12-item patient reported outcome measure specifically developed to assess the outcomes of elbow surgery. It is the only PROM for elbow surgery that has been tested in a surgical context and shown to be reliable, valid, and responsive. Recovery and general improvement following elbow surgery can be indicated by OES. Also, it can be used for nonsurgical treatments such as physiotherapy, injections, and joint supplements. OES has three domains with four questions in each. Elbow function, pain, and social-psychological domains record the patient's opinion about the elbow and its impact on quality of life [13].

### 2.5. Disabilities of the Arm, Shoulder, and Hand (DASH)

The DASH questionnaire is a 30-item patient-rated measure designed to assess physical function and symptoms in patients with any form of musculoskeletal disorder of the upper limb. The questionnaire was designed to help describe the disability experienced by people with upper limb disorders and to monitor changes in symptoms and function over time [14]. The Persian version of DASH was validated and cross-culturally adapted by Mousavi et al. and has been used in many studies up to present [15].

### 2.6. Short Form 36 Health Survey (SF-36)

The SF-36 is a questionnaire of generic, coherent, and easily administered quality of life measurement that includes an 8-scale profile of functional health and psychometrical health measures. Physical function (PF), role-physical (RP), bodily pain (BP), and general health (GH) are the four subscales of functional health measures. Vitality (VT), social function (SF), role-emotional (RE), and mental health (MH) are four subscales of psychometrical health. The SF-36 has proven useful in surveys comparing the relative burden of diseases and in differentiating the health benefits produced by a wide range of different treatments. Among the most frequently studied diseases and conditions that apply SF-36 are arthritis, back pain, osteoarthritis, spinal injury, and trauma [1, 16]. The Persian version of SF-36 has been validated by Montazeri et al. in 2005 and Jafari et al. in 2008 [17, 18].

## 3. Results

The overall Cronbach's *α* coefficient was 0.92. The Cronbach's *α* for function, pain, and social-psychological subscales of the OES was 0.95, 0.86, and 0.85, respectively, showing good to excellent internal consistency (Table 1). The overall ICC was 0.85 and it was 0.90, 0.76, and 0.75 for function, pain, and social-psychological subscales, respectively (Table 1).

The correlation coefficient between OES and the DASH was 0.80 showing strong correlation. The correlation between OES and most of the subscales of the SF-36 was high, significant, and in a reverse direction showing moderate to strong correlation of the Persian OES with other measures in the same context (Table 2).

## 4. Discussion

According to above-mentioned data, the Persian OES showed good test-retest reliability and excellent internal consistency. Also it is confirmed to be a valid instrument to be applied in patient after elbow interventions.

In our study, the Persian OES reliability was checked by test-retest calculation and Cronbach's alpha coefficient and each of these two manners of calculation revealed excellent reliability for the Persian OES. Currently, English, Dutch, and Danish versions are available as validated versions of the OES [7, 8, 13]. In a study by Dawson et al. in 104 patients who underwent elbow surgery in the UK, the OES questionnaire was analyzed for validity, reliability, and responsiveness [13]. In this study, the Cronbach's alpha for function, pain, and socio-psychological domains was 0.90, 0.89, and 0.84, respectively. The ICC values for each domain were 0.89, 0.98, and 0.87, respectively. de Haan et al. validated and adapted the OES to the Dutch language in 69 patients in 2011 [7]. Cronbach's alpha coefficient for each of the three domains of the Dutch OES was 0.90, 0.87, and 0.90, respectively. In 2013, Plaschke et al. published their study on validation and cross-cultural adaptation of the Danish version of the OES [8]. A total of 130 patients with a prior history of total elbow arthroplasty during 1981 to 2008 participated in their study. Patients in this study also completed DASH and MEPI questionnaires. Overall, the alpha test was 0.99 and the test-retest reliability coefficient was 0.998, which are more than the values in our study. Internal consistency coefficient for function, pain, and social-psychological domains was 0.998, 0.996, and 0.996, respectively.

In our study, the Spearman's correlation coefficient of the Persian OES with DASH and SF-36 was statistically significant, except for in the social function (SF) domain of the SF-36. The SF-36 is a general measure, which is less affected by local problem than a region-specific measure such as OES [19, 20]. A difference in responsiveness is predictable and correlation coefficients would be lower when compared to the DASH, which is a more specific measure than the SF-36 but more general measure than OES. Hence, the direction of changes and significance level will reveal that it measures what it is supposed to measure in the same context. In the Dutch version of the OES, validity of the test was also checked with the Quick-DASH, VAS, and MEPI. In De Haan's study, the Spearman's correlation coefficient of function, pain, and social-psychological domains was −0.43, −0.44, and −0.47 with the Quick-DASH; −0.33, −0.38, and −0.42 with VAS; and 0.68, 0.77, and 0.77 with MEPI, respectively. In the Danish OES, Pearson's correlation coefficient for function, pain, and socio-psychological domains was 0.78, 0.81, and 0.80 with MEPI and −0.66, −0.49, and −0.58 with DASH, respectively.

A limitation to our study is that we administered this questionnaire to patients who had the history of elbow injection or surgery with no time limitation. This may change the responsiveness between patients with recent surgeries and patients with old surgeries. Another limitation of our current study is the validation of the Persian OES with two general health related questionnaires because, until now, the available Persian-translated scoring systems have been the DASH and SF-36 and the only elbow-relevant scoring system used in the current study was the Persian-DASH and the SF-36 is a general health status scoring system. In De Haan and Plaschke's studies, the translated versions were validated with at least two relevant scoring systems such as the MEPI score, which is one of the most commonly used physician-based elbow rating systems [7, 8]. DASH and SF-36 are patient-based questionnaires and using patient-based scorings is one of the advantages of this study.

Oxford Elbow Score is a patient reported measure that can be filled individually. The simplicity of completion and scoring makes this questionnaire applicable to let the patient interpret the results and to have an active role in decision-making for the next step in the treatment process.

## 5. Conclusion

The Oxford Elbow Score proved to be a valid and reliable instrument to be applied after interventions about the elbow. It can be used for research purposes in order to follow the recovery of patients.

## Supplementary Material

File. Validated Persian Oxford Elbow Score (P-OES). We used the 10-step forward-backward method following the Wild et al. guideline to translate the OES into Persian.

## Figures and Tables

**Table 1:** Internal consistency and test-retest reliability of the Persian version of the Oxford Elbow Score.

Subscale	Number of items	Cronbach's alpha	(Intraclass correlation coefficient)		
			ICC	95% CI	*P* value
Function	4	0.95	0.9	0.80–0.95	<0.001
Pain	4	0.86	0.76	0.56–0.88	<0.001
Social-psychological	4	0.85	0.75	0.54–0.87	<0.001

Total	12	0.92	0.85	0.71–0.92	<0.001

ICC: intraclass correlation coefficient and CI: confidence interval.

**Table 2:** Convergent validity expressed by Spearman's correlation (*r*) between subscales of Persian OES and the DASH and SF-36 (*N* = 92).

	Oxford Elbow Score			
	Function	Pain	Social-psychological	Total
SF-36				
PCS	(−0.63)∗∗	(−0.56)∗∗	(−0.61)∗∗	(−0.67)∗∗
Physical functioning	(−0.58)∗∗	(−0.48)∗∗	(−0.49)∗∗	(−0.58)∗∗
Role physical	(−0.48)∗∗	(−0.39)∗∗	(−0.55)∗∗	(−0.53)∗∗
Bodily pain	(−0.66)∗∗	(−0.76)∗∗	(−0.72)∗∗	(−0.80)∗∗
General health	(−0.37)∗∗	(−0.34)∗∗	(−0.38)∗∗	(−0.41)∗∗
MCS	(−0.37)∗∗	(−0.44)∗∗	(−0.49)∗∗	(−0.48)∗∗
Vitality	(−0.44)∗∗	(−0.43)∗∗	(−0.48)∗∗	(−0.50)∗∗
Social functioning	(−0.14)	(−0.19)	(−0.24)∗	(−0.21)∗
Role emotional	(−0.51)∗∗	(−0.39)∗∗	(−0.55)∗∗	(−0.54)∗∗
Mental health	(−0.31)∗∗	(−0.47)∗∗	(−0.39)∗∗	(−0.43)∗∗
DASH	0.77∗∗	0.64∗∗	0.73∗∗	0.80∗∗

PCS: physical condition scale and MCS: mental condition scale.

∗∗Correlation is significant at the 0.01 level (2-tailed).

∗Correlation is significant at the 0.05 level (2-tailed).
