# Supplementary material for: Validity and Cross-Cultural Adaptation of the Persian Version of the Oxford Elbow Score
Source: Int J Rheumatol. 2014 Aug 26;2014:381237. doi: 10.1155/2014/381237 (PMC4158467; doi:10.1155/2014/381237)
Supplement: Supplementary file 1 — File. Validated Persian Oxford Elbow Score (P-OES). We used the 10-step forward-backward method following the Wild et al. guideline to translate the OES into Persian. [file 381237.f1.pdf]

Oxford Elbow Score

نام بیمار: سن : تحصیلات:

آدرس: شماره همراه و ثابت:

شغل: سمت درگیر:

۱. در طی ٤ هفته گذشته آیا شما مشکلی در زمینه بلند کردن اجسام در خانه داشته اید ؟ (نظیر بیرون گذاشتن زباله ها)

☐ هیچ مشکلی نداشته ام

☐ مختصری مشکل داشته ام

☐ در حد متوسط مشکل داشته ام

☐ بسیار دشوار بوده است

☐ غیر قابل انجام بوده است

۲. در طی ٤ هفته گذشته آیا بدلیل مشکلات آرنج، حمل کردن کیسه های خرید از فروشگاه برایتان سخت بوده است؟

☐ نه ـ مشکلی نداشته ام

☐ تا حدی برایم سخت است

☐ گهگاه برایم دشوار است

☐ بسیار سخت است

☐ غیر قابل انجام است

۳. آیا در طی ٤ هفته گذشته بدلیل درد و مشکلات آرنج، انجام کارهای بهداشتی و حمام کردن برایتان مشکل بوده است؟

☐ خیر ـ مشکلی نداشته ام

☐ مختصری مشکل داشته ام

☐ در اکثر اوقات مشکل داشته ام

☐ برایم بسیار مشکل بوده است

☐ غیر قابل انجام بوده است

٤. در طی ٤ هفته گذشته آیا بدلیل مشکلات آرنج، در لباس پوشیدن مشکلی داشته اید؟

☐ خیر ـ مشکلی نداشته ام

☐ مختصری برایم مشکل است

☐ در اکثر اوقات مشکل دارم

☐ بسیار برایم سخت است

☐ غیر قابل انجام است

۵. در طی ٤ هفته گذشته آیا احساس کرده اید که مشکلات آرنج زندگیتان را تحت تأثیر قرار داده است؟

☐ خیر ـ به هیچ وجه

☐ گهگاه

☐ بعضی روزها

☐ اکثر روز ها

☐ هر روز

٦. مشکلات و درد آرنجتان تا چه حد فکر و ذهن شما را مشغول کرده است؟

☐ گهگاه ـ بسیار کم

☐ تا حدی ـ بعضی اوقات

☐ اکثر اوقات

☐ همیشه

۷. آیا در طی ٤ هفته گذشته درد شبانه آرنج داشته اید؟

☐ نه ـ نداشته ام

☐ یک یا دو شب

☐ بعضی شب ها

☐ اکثر شب ها

☐ هر شب

۸. تا چه حدی درد آرنجتان مزاحم خواب شماست؟

☐ به هیچ وجه

☐ گهگاه

☐ بعضی روزها

☐ اکثر روز ها

☐ هر روز

۹. تا چه حد مشکل آرنجتان در کارهای روزمره تان اختلال ایجاد می کند؟ (کارهای روزمره تان را مختل کرده است)

☐ به هیچ وجه تأثیری نداشته است

☐ مختصری مختل شده است

☐ در حد متوسط مختل شده است

☐ بسیار زیاد مختل شده است

☐تماماً مختل است

۱۰. آیا مشکل آرنج تان برای شرکت شما در فعالیت های تفریحی مانع ایجاد کرده است؟

☐ به هیچ وجه تأثیری نداشته است

☐ مختصری مختل شده است

☐ در حد متوسط مختل شده است

☐ بسیار زیاد مختل شده است

☐تماماً مختل است

۱۱. بدترین دردی که در آرنج داشته اید را چگونه توصیف می کنید؟

☐ درد نداشته ام

☐ در حد مختصر

☐ در حد متوسط

☐ درد شدید

☐ غیر قابل تحمل بوده است

۱۲. دردی را که معمولاً در آرنج دارید، چگونه توصیف می کنید؟ (درد آرنجتان در چه حدی بوده است)

☐ درد نداشته ام

☐ درد مختصر

☐ درد متوسط

☐ درد شدید

☐ غیر قابل تحمل

دانشگاه علوم پزشکی مشهد

مرکز تحقیقات ارتوپدی
Orthopedic Research Center

با تشکر از همکاری شما
